# Supplementary material for: Post hurricane Harvey dataset: Portable free fall penetrometer and chirp sonar measurements of Texas rivers
Source: Data Brief. 2022 Apr 22;42:108203. doi: 10.1016/j.dib.2022.108203 (PMC9065586; doi:10.1016/j.dib.2022.108203)
Supplement: Supplementary file 1 [file mmc1.docx]

**Post Hurricane Harvey dataset: Portable Free Fall Penetrometer and Chirp Sonar Measurements of Texas Rivers**

**Authors**

Reem Jaber^1^, Nina Stark, Ph.D., M.ASCE^2^, Navid Jafari, Ph.D., M.ASCE^3^, Nadarajah Ravichandran, Ph.D., M.ASCE^4^

**Affiliations**

^1^ Ph.D. Candidate, Department of Civil and Environmental Engineering, Virginia Tech., 200 Patton Hall, Blacksburg, VA 24061. e-mail: reemj@vt.edu

^2^ Associate Professor, Department of Civil and Environmental Engineering, Virginia Tech., 200 Patton Hall, Blacksburg, VA 24061. e-mail: ninas@vt.edu

^3^ Assistant Professor, Department of Civil and Environmental Engineering, Louisiana State University, 3212D Patrick F. Taylor Hall, Baton Rouge, LA 70803. e-mail: [njafari@lsu.edu](mailto:njafari@lsu.edu)

^4^ Associate Professor, Department of Civil Engineering, Clemson University, 202 Lowry Hall, [Clemson, SC 29634](mailto:Clemson,%20SC%2029634). e-mail: [nravic@clemson.edu](mailto:nravic@clemson.edu)

**Corresponding author(s)**

Reem Jaber (reemj@vt.edu)

**Abstract**

This data article includes datasets collected at three sections of the Guadalupe River, Brazos River, and Colorado River in Texas, USA, almost ten months post Hurricane Harvey. Instruments used include a Portable Free Fall Penetrometer (PFFP), Chirp Sonar, Side Scan Sonar (SSS), Acoustic Doppler Current Profiler (ADCP) and sediment grab sampler. Measurements were collected from small vessels such as canoes and a 6-feet inflatable zodiac and were supported by long term hydrodynamic data from local river water level and discharge gages. Laboratory testing performed on samples collected included grain size analysis, Atterberg test, and erodibility testing using an Erosion Function Apparatus (EFA). Data collected were analyzed to estimate sediment strength derived from the PFFP, backscatter intensity recorded by the chirp sonar, and soil sample characteristics. The dataset includes raw and processed data for the measurements recorded by the instruments, location of measurements, and laboratory testing grouped for each river with a readme file which gives a potential for reuse by other researchers for further analysis if needed. This data article is representing supplementary data to the following research article published in Engineering Geology [1]:

Jaber, R., Stark, N., Jafari, N., & Ravichandran, N. “Combined Portable Free Fall Penetrometer and Chirp Sonar Measurements of three Texas River Sections Post Hurricane Harvey.”

Raw data was published [2]: Stark, N. Jafari, N. Ravichandran, R. Jaber, R. (2020). Combined Geotechnical and Geophysical Investigation of Texas Rivers Post Hurricane Harvey. in Combined Geotechnical and Geophysical Investigation of Texas Rivers Post Hurricane Harvey. DesignSafe-CI. <https://doi.org/10.17603/ds2-835m-zp94>

**Keywords**

Offshore geotechnical properties, site characterization, riverine environments, extreme events, geotechnical investigation

**Specifications Table**

| **Subject** | Civil and Structural Engineering |
| --- | --- |
| **Specific subject area** | Geotechnical and geophysical site characterization; riverine sediment dynamics. |
| **Type of data** | Image  Chart  Graph  Figure |
| **How data were acquired** | The data was collected through a field survey using the instruments mentioned below, in addition to soil testing conducted on sediment samples collected.  Instruments: portable free fall penetrometer, chirp sonar, side scan sonar, acoustic doppler current profiler, erosion function apparatus, sieve shaker, Atterberg apparatus, MATLAB, Excel spreadsheets |
| **Data format** | Raw  Analyzed |
| **Parameters for data collection** | Data were collected using small vessels and under active river flow, which limited the navigational abilities and led to spatial uncertainty regarding the measurement locations of the PFFP and chirp sonar on the order of meters. Chirp sonar was kept on low energy mode as the water levels approached the minimal water depth feasible for the system. Shoals also led to some disruptions in data collection. The chirp sonar was attached to the vessel side, and the PFFP was deployed by hand.  Samples collected during the survey were limited to surficial soil due mostly to the sandy nature of the soil and sampling methods. |
| **Description of data collection** | Data was collected in sections of the Guadalupe, Brazos, and Colorado rivers in July 2018, nine months post Hurricane Harvey. Instruments listed above were deployed from small vessels and were supported by long term hydrodynamic data from local river water level and discharge gages. |
| **Data source location** | City/Town/Region: Guadalupe River, Brazos River, Colorado River-Texas  Country: United States  Latitude and longitude (and GPS coordinates, if possible) for collected samples/data: Guadalupe River: 28˚45'06.89" N, 97˚00'24.78" W; Brazos River: 29˚34'20.99" N, 95˚41'51.81" W; Colorado River: 28˚59' 02.41" N, 96˚00'01.28" W |
| **Data accessibility** | The data is published in a public repository [2].  Repository name: Design Safe-CI  Data identification number: 10.17603/ds2-835m-zp94  Direct URL to data: <https://doi.org/10.17603/ds2-835m-zp94>  Stark, N. Jafari, N. Ravichandran, R. Jaber, R. (2020). Combined Geotechnical and Geophysical Investigation of Texas Rivers Post Hurricane Harvey. in Combined Geotechnical and Geophysical Investigation of Texas Rivers Post Hurricane Harvey. DesignSafe-CI. <https://doi.org/10.17603/ds2-835m-zp94> |
| **Related research article** | [1]: R. Jaber, N. Stark, N. Jafari, N. Ravichandran, Combined Portable Free Fall Penetrometer and Chirp Sonar Measurements of three Texas River Sections Post Hurricane Harvey, J. Engineering Geology 294 (2021): 106324. <https://doi.org/10.1016/j.enggeo.2021.106324> |

**Value of the Data**

- The data was collected post Hurricane Harvey ,representing measurements of sections identified in the Guadalupe, Brazos, and Colorado Rivers, which are known for their active sediment dynamics and erosion. These processes impose risks on the stability of infrastructure, but measurements can be challenging while water levels are still elevated. This dataset offers data collection strategies for gaining insights into sediment dynamics during a storm.
- This dataset is widely applicable to several research topics and fields. This includes correlation of geotechnical and geophysical instruments; post-storm surveying; riverine sediment properties; scour and erosion which can be of interest to oceanographers, marine scientists, geophysicists, coastal engineers, and others.
- The available data include raw and processed data. Researchers might exploit processed data for further analysis as a first step to accomplish other research goals, particularly in simulations studying sediment dynamics and scour. Raw data can also be utilized with different processing approaches, which can optimize the use of data collected and data collection strategies.

**Data Description**

Figures:

- Figure S1: Water levels of the Guadalupe River near Victoria, TX in 2017 collected by the USGS gage 08176500 [3]. The data includes water levels recorded during Hurricane Harvey on August 31^st^.
- Figure S2: Google Earth (2018) image of the Brazos River close to Sugarland, Texas 29°34'21.61" N, 95°41'51.47" W (Map data: Google, SIO, NOAA, US Navy, NGA, GEBCO). Fig. S2(a) shows an overview image of the location, and Fig.S2(b) shows the survey site with PFFP (*Bluedrop*) deployment locations along transect 1 (blue circles), transect 2 (red circles) and sediment sampling locations (white circles). Deployments in the Guadalupe River (28˚45'06.89" N 97˚00'24.78" W) were divided into 7 transects around the piles of the E Frontage Rd Bridge., and deployments in the Colorado River (28˚59'02.41" N 96˚00'01.28" W) were divided into 5 transects around the bridge pillars. For both rivers, samples were collected in the vicinity of riverbanks.
- Figure S3: Change in the location of riverbanks in the investigated section of the Brazos River between January 2009 (yellow lines), August 2017 (red lines), and February 2019 (Map data: Google, SIO, NOAA, US Navy, NGA). The images show the progressive erosion along the riverbanks close to the study area during the last decade, with erosion reaching up to ~44 m in some locations.
- Figure S4: Color coded quasi-static bearing capacity *qsbc* ranges at Guadalupe River (triangular shaped deployments were done on July 16th; circular shaped deployments were done on July 17th). The deployments were grouped and divided into six categories based on the *qsbc* values recorded. More variations in the *qsbc* values were observed along the western riverbank, close to the downstream bridge pier.
- Figure S5: Three panels of the measured pressure responses. (a) represents Type A, (b) represents Type B, and (c) represents Type C prior to penetration of the riverbed (positive distance), during, and after penetration (negative distance) into the riverbed. The black solid line represents the recorded pressure, the red line represents a smoothened recorded pressure, and black dashed line represents the projected hydrostatic increase with depth starting from the riverbed surface. PWP responses among rivers were grouped and divided based on the three types. Type A profiles were characterized by sub-hydrostatic pressures just before and during penetration, type B profiles deviated from the hydrostatic projection during penetration and changed towards supra-hydrostatic pressures, and type C profiles were identical to the projected hydrostatic pressure and supra-hydrostatic during penetration.
- Figure S6: Variations of (a) Normalized backscatter intensity NBI and qsbc and (b) water depth recorded by PFFP and chirp with distance along transect 1 (blue color) and transect 2 (red color) at Brazos River. The values of NBI and qsbc agreed well from the river center towards the eastern riverbank, where the soil type is considered predominantly sandy, whereas a significant mismatch was observed towards the western riverbank.

Other figures, codes and excel spreadsheets are described in the readme file published in the public data repository.

**Experimental Design, Materials and Methods**

All measurements were performed from small vessels (canoes and a 2.5 m inflatable zodiac). Some navigational limitations were encountered due to the presence of debris leading to small deviations from the planned transects. Deployments were grouped and divided into six transects at the Guadalupe River which included cross-river, along-river transects, and measurements around the piles of the E Frontage Rd bridge. For the Brazos River, a total of 33 deployments were distributed along two transects: Transect 1 is orientated across the river and Transect 2 is a short section along the western riverbank (Fig. S2). For the Colorado River, a total of 49 PFFP deployments were conducted along 5 transects around the bridge piers. Samples for the Erosion Function Apparatus test were collected from the riverbanks for each of the rivers mentioned. Each of the deployed instruments is described in detail in this section with further details provided in the related research article (Jaber et al. 2021).

Portable free fall penetrometer (PFFP):

The PFFP has five accelerometers that continuously record decelerations. It free falls through the water column until it hits the riverbed. The advancement through the riverbed depends primarily on the soil resistance against the probe 4]. Buoyancy in water is considered while additional impacts of soil buoyancy have been found negligible. Literature have also addressed the contribution of drag force to the total resistance measured by the PFFP, which was also neglected due to the shallow penetration depth and the penetrometer shape [5]. Therefore, soil bearing resistance is assumed the dominant force leading to penetrometer deceleration in the riverbed, enabling a simple relationship between deceleration and soil bearing resistance force through Newton’s second law.

The ultimate dynamic bearing capacity (*q_ud_*) resisting the probe can then be estimated using the soil bearing resistance force (*F*) over the surface area (*A*) subjected to load, as follows:

$q_{ud}=\frac{F}{A}$

The high (in relation to typical geotechnical in-situ testing) and dynamically changing penetration velocity of the PFFP is expected to lead to changes in the ultimate dynamic bearing capacity due to strain rate effect. Hence, the application of a strain factor is required to derive consistent data unaffected by changes in penetration velocity. The approach suggested by Dayal and Allen [3] is used here to correct for strain rate effects, resulting in an equivalent quasi-static bearing capacity of the soil *(qsbc)*. The *qsbc* value simulates the soil bearing capacity at a constant penetration velocity, commonly taken as 2 cm/s, which reflects the standard cone penetration test penetration velocity.

There are various forms for strain rate factor in literature, and the issue is also still subject to research regarding portable free fall penetrometers. Here, the approach initially presented by [6] and then suggested for portable free fall penetrometers on sand by [7] was applied:

$f=1+Klog \left( \frac{v_{dyn}}{v_{o}} \right)$

where $\nu_{dyn}$is the dynamic penetration velocity of the penetrometer, and $v_{o}$ is the reference penetration velocity set at 2 cm/s. *K* is a dimensionless soil-dependent factor and can affect the PFFP *qsbc* values significantly. Different values have been used in literature [8] as there is still no general agreement regarding the best choice of *K* values for sands. However, a range between 1 and 1.5 has been suggested in literature for impact velocity > 3 m/s and particularly when fines can be present [8]. Due to lack of more information regarding the soil type, and the difficulties associated with sample collection, limited information was available on the choice of *K.* Limitations in sample collection hindered sample testing that could calibrate or validate *K* values with the site specific soil type. Therefore, based on literature and for lack of better knowledge, a range of *K* was assumed between 1 and 1.5. An estimate of quasi-static bearing capacity (*qsbc*) is then derived as follows:

$qsbc=\frac{q_{ud}}{f}$

The pressure transducer in the PFFP records pore pressure up to 2 MPa. However, the pressure recorded by the pressure sensor during the free fall of the penetrometer through the water column is less than the hydrostatic pressure due to Bernoulli’s effect [9]. Therefore, a correction must be applied to calculate the correct water depth from pressure measurements:

$$h_{c}=h_{u}+\frac{v_{i}^{2}}{g}$$

where *h_u_* is the uncorrected water depth (m) from the recorded pressure at impact, *v_i_* is the impact velocity of the PFFP (m/s), and *h_c_* is the corrected water depth (m).

Chirp sonar:

The chirp sonar is mounted to the side of the boat. The sound transmitted from the chirp sonar is reflected off seafloor sediment layers based on the different acoustic properties of each layer. The strength of the reflected signal and the time needed to reach the source/receiver is used to locate the depth of the layers, and based on that, display an image of the riverbed stratigraphy [10]. The specific device can be used for marine geophysical surveys of up to 150 m of water depth with a transmit pulse rate from 4 to 10 Hz and the frequency is 10 kHz. It has a blanking distance (i.e., start distance that is affected by proximity to the transducer) of approximately 1 m which limits measurements in shallow water depths. The post-processing in the acoustic dataset presented here is limited to the depicting of different riverbed layers according to the strength of the reflected signal using the manufacturer’s software. The data measured was stored in seg-y files that can be replayed in the StrataBox HD. Images were extracted from replays. Backscatter values were extracted at specific locations and the output of the chirp return signal is represented in the form of the normalized backscatter intensity (*NBI*), as a percentage of the maximum strength achieved by the return signal. These *NBI* values were chosen at similar locations with PFFP deployments with some uncertainty in the order of meters.

$$NBI=\frac{BI}{\max BI}*100$$

Erosion Function Apparatus (EFA):

Samples collected from riverbanks were tested using the erosion function apparatus (EFA). This setup introduced by Briaud et al. [11]measures the erodibility of soil under various shear stresses due to the water flow at controlled velocities. A tube containing the collected soil sample is placed underneath the water conduit where water flow velocity can be controlled. The flow velocity created a shear stress on the soil surface and if the shear stress applied on the soil sample surface exceeds the critical shear stress, erosion is initiated, and the erosion volume can be measured over specific time for given shear stresses. The measurements are presented as erosion rate per flow velocity or shear stress and classified into five erodibility levels based on the erosion volume ranging from non-erosive to very high erodibility. EFA tests have been performed on samples from the Guadalupe, Brazos, and Colorado River with flow velocities ranging from 0.2 m/s to 5.6 m/s.

**Acknowledgments**

The authors would like to acknowledge the National Science Foundation for funding the research and work presented through grant CMMI-1822307. The authors express gratitude to Julie Paprocki, Dennis Kiptoo, Matthew Florence, and Brian Harris for their data collection efforts. The authors would also like to thank Jean-Louis Briaud and Iman Shafii for assistance with the EFA testing.

**Declaration of Competing Interest**

The authors declare that they have no known competing financial interests or personal relationships which have or could be perceived to have influenced the work reported in this article.

**Ethics Statement**

Not applicable

**Credit Author statement**

**Reem Jaber:** Visualization, Methodology, Formal analysis, Validation, Investigation, Data Curation, Writing - Original Draft, Writing - Review & Editing

**Nina Stark:** Conceptualization, Methodology, Formal analysis, Investigation, Data Curation, Writing - Review & Editing, Supervision, Project administration, Funding acquisition

**Navid Jafari:** Conceptualization, Investigation, Writing - Review & Editing, Supervision, Project administration, Funding acquisition

**Nadarajah Ravichandran:** Conceptualization, Investigation, Writing - Review & Editing, Supervision , Project administration , Funding acquisition

**References**

[1] R. Jaber, N. Stark, N. Jafari, N. Ravichandran, Combined Portable Free Fall Penetrometer and Chirp Sonar Measurements of three Texas River Sections Post Hurricane Harvey, J. Engineering Geology 294 (2021) 106324. https://doi.org/10.1016/j.enggeo.2021.106324.

[2] N. Stark, N. Jafari, N. Ravichandran, R. Jaber, Combined Geotechnical and Geophysical Investigation of Texas Rivers Post Hurricane Harvey. in Combined Geotechnical and Geophysical Investigation of Texas Rivers Post Hurricane Harvey. DesignSafe-CI,2020. <https://doi.org/10.17603/ds2-835m-zp94> .

[3] National Water Information System, USGS Current Conditions for the Nation-Station 08114000. https://nwis.waterdata.usgs.gov/nwis/uv?, 2019 (accessed 10 June 2019).

[4] C. Bilici, N. Stark, C.T. Friedrichs, G.M. Massey, Coupled sedimentological and geotechnical data analysis of surficial sediment layer characteristics in a tidal estuary, J. Geo-Marine Letters, 39.3(2019) 175-189. https://doi.org/10.1007/s00367-019-00565-3.

[5] N. Stark, B. Radosavljević, B.M. Quinn, H. Lantuit, Application of a portable free-fall penetrometer for the geotechnical investigation of the Arctic nearshore zone, Canadian Geotechnical Journal, 54.1(2017) 31–46. https://doi.org/10.1139/cgj-2016-0087.

[6] U. Dayal, J.H. Allen, Instrumented impact cone penetrometer, Canadian Geotechnical Journal, 10.3(1973), 397-409. https://doi.org/10.1139/t73-034.

[7] N. Stark, G. Coco, K.R. Bryan, A. Kopf, In-situ geotechnical characterization of mixed-grain-size bedforms using a dynamic penetrometer, J. of Sedimentary Research, 82.7(2012) 540-544. <https://doi.org/10.2110/jsr.2012.45>.

[8] R.D. Stoll, Y.F. Sun, I. Bitte, Seafloor properties from penetrometer tests, J. of Oceanic Engineering, 32.1(2007) 57-63. <https://10.1109/JOE.2007.890943>.

[9] G. Lucking, N. Stark, T. Lippmann, S. Smyth, Variability of in situ sediment strength and pore pressure behavior of tidal estuary surface sediments, J. Geo-Marine Letters, 37.5(2017) 441-456. <https://doi.org/10.1007/s00367-017-0494-6>.

[10]SyQwest, StrataBox HD Chirp Operations and Maintenance Manual, http://www.syqwestinc.com/products/sub-bottom-profilers/stratabox (accessed June 5, 2019).

[11] J.L. Briaud, F. Ting, H.C. Chen, Y. Cao, S.W. Han, K. Kwak, Erosion Function Apparatus for Scour Rate Predictions, J. of Geotechnical and Geoenvironmental Engineering, 127.2(2001) 105-113. https://doi.org/10.1061/(ASCE)1090-0241(2001)127:2(105).
